# Emergency medicine in Scandinavia - an outstanding opportunity for research

**DOI:** 10.1186/1757-7241-18-5

**Published:** 2010-01-29

**Authors:** Mikkel Brabrand, Ulf Ekelund

**Affiliations:** 1 Medical Admission Unit 272, Sydvestjysk Sygehus Esbjerg, Denmark; 2 Department of Clinical Science at Lund, Lund University, Skåne University Hospital, Sweden

## Editorial

It was not long ago that emergency medicine (EM) was only something for the rest of the world. Emergency patients in the Scandinavian countries were still being handled by young and poorly trained doctors from many specialties and no doctor tended especially to the undiagnosed patient in the emergency department (ED). The work environment was hostile, and the nurses had to make do with temporary physicians who did nothing to improve it. In Norway doctors from different specialities ran the ED, in Denmark it more or less was managed by the orthopaedic surgeons and in Sweden general surgeons were often in charge, but few doctors really took ownership of the ED. However, in 1999 the Swedish Society for Emergency Medicine (SWESEM) was established and things started to change. The Danish Society for Emergency Medicine (DASEM) was established in 2006 and the work to improve the care of emergency patients began. Finland also has a society but no speciality, whereas in Norway there seem to be no plans to establish EM as a specialty. Sweden is the country that has now come the longest way in implementing emergency medicine and Denmark is following close behind. In Sweden, the EDs are now starting to be populated by designated doctors working only there, and specialists in EM are increasing. In Denmark the National Board of Health has recently proposed major changes in the organisation of the entire emergency care system. This will lead to a completely new way of organising the EDs and admission units at most hospitals in Denmark.

As EM is still in its infancy in our region of the world, there is an outstanding opportunity for conducting research. Now, before changes are implemented in all EDs, is *the *chance to design before versus after studies, and several important fields of research are opening up.

First, we believe the implementation of EM as a specialty is a good idea, but we basically don't know if this is true. Evidence-Based Medicine is now the universal credo for all specialties, but no physician speciality in medicine was originally established based on solid evidence. Nor will this be the case for EM, but we now have a rare chance to test whether EM as a speciality and a higher level of competence in the ED makes a difference. There is evidence from previous studies that emergency physicians are at least as competent as their counterparts from the established specialities [1]. Emergency physicians improve the time to revascularization of patients with acute coronary syndrome, interpret ECG's equally to - or even better than - residents in internal medicine and are equally good at treating cardiac or respiratory arrest as others. In Scandinavia we now have a chance to design studies on the effect of EM specialists on the quality of care, on patient flow and on throughput in the ED. Will EM specialists reduce mortality, morbidity and the cost of treatment, and will they improve patient safety?

Second, which ED organisation is best? Hospital owners are using billions of Kroner on building new hospitals and refurbishing existing EDs. But how much do we know on how to design and run these departments? Not much! Should they contain their own observation units for in-patients? Should the specialist in EM greet the patient at the door or should it be a nurse? Or could perhaps a secretary do it equally well? Should we do triage or is streaming the answer? Is the goal of a four hour maximum stay in the ED well founded? Can these large amounts of money be used better? Would it be better to invest in physician training than in buildings? Perhaps the improvement of the in-hospital organisation of emergency care provides no benefit to the patients? Let's use this golden opportunity to find out!

Third, EDs differ from other departments in the need for specialist coverage around the clock. Acutely ill patients need optimal care and treatment, whatever the time. This is one of the major hallmarks of EM; the Emergency Physician is always available, 24 hours a day, seven days a week. However, what is the optimal organization of the work force during the night? What will reduce physician burn-out and slip-ups in patient management? Perhaps changing the working hours of physicians can help? In this context, Croskerry and Sinclair [2] has suggested staffing schedules similar to those used in casinos (where staff also needs to be on their toes 24 hours a day). During the implementation of EM in Scandinavia, we have a chance to elucidate this.

In addition to the current window of opportunity, we believe that Scandinavian hospitals (as well as ambulances) generally offer a tremendous research environment. Computerized patient records are everywhere, personal identification numbers are used throughout hospitals and society, and co-operation is relatively easy due to few health care principals. Because of this, the countries are also some of the 'promised lands' for health care registries; in Sweden alone there are at least 70 national registries. In fact, SWESEM has taken the initiative to start a national emergency care registry which is now collecting its first data.

Despite these good conditions, performing EM research in Scandinavia will not be entirely uncomplicated, at least for the time being. As EM is a new area of interest it has to compete with the established specialties for financial support, and in both Denmark and Sweden many researchers have difficulties financing their projects. Traditional grants are difficult to get, and EM is not an area of great focus from the medical industry. As a result DASEM has experimented with microfinance in EM [3], but this is a solution only for minor projects. The major projects need to find other financing, which is often hard. We think it is reasonable to believe however, that as our research groups get established and if the value of EM becomes clear, national or regional grant bodies will acknowledge the need for adequate funding for EM research. In the mean time we have to make do with what we have, and perhaps also collect our own money. A Scandinavian research fund for EM could be a solution, and small steps in this direction have already been taken in Sweden.

So we have an almost perfect opportunity for research and we have the right environment. The financial problems are often solvable, and will hopefully become less with time. Why wait? Let's get started.

Mikkel Brabrand and Ulf Ekelund

Section editors, Emergency Medicine, Scandinavian Journal of Trauma, Resuscitation and Emergency Medicine
